# The Prognostic Significance of Spliceosomal Proteins for Patients with Glioblastoma

**DOI:** 10.1134/S1607672922020090

**Published:** 2022-05-10

**Authors:** T. D. Larionova, T. F. Kovalenko, M. I. Shakhparonov, M. S. Pavlyukov

**Affiliations:** Shemyakin–Ovchinnikov Institute of Bioorganic Chemistry, Russian Academy of Sciences, Moscow, Russia

**Keywords:** glioma, glioblastoma, RNA splicing, patient survival prognosis

## Abstract

Glioblastoma (GBM) is considered one of the most aggressive human cancers. Earlier, our group have demonstrated that alternative RNA splicing plays an important role in the regulation of the GBM phenotype. To continue this study, we analyzed the type of RNA splicing and the expression levels of the spliceosomal genes in a large number of tumor tissue samples and patient-derived GBM sphere lines. We demonstrated that the expression level of splicing factors allows dividing GBM patients into groups with different survival prognosis and also reflects the phenotype of the tumor. In addition, we identified the alternative splicing events that may regulate the GBM phenotype. Finally, we for the first time compared the expression profiles of the spliceosomal genes in different regions of the same tumor and identified splicing factors whose expression most significantly correlates with GBM patients’ survival. Aforementioned data emphasize the important role of pre-mRNA splicing in GBM progression.

Glioblastoma is the most common primary brain tumor in adults. Despite the enormous efforts aimed at combating this disease, the existing methods of treating glioblastomas are mainly of a palliative nature and can increase the average patient survival from 3 months (without treatment) to 2 years (provided maximum surgical removal of the tumor followed by chemotherapy and radiotherapy) [1, 2]. Such a negative outcome is due to the extreme intra- and intertumor heterogeneity. For example, based on genetic mutations, glioblastoma cells are divided into IDH-mutant and wild-type IDH [3]; based on DNA methylation, tumors are divided into CIMP+ and CIMP– [4]; finally, according to the level of gene expression, glioblastomas can belong to the classical, mesenchymal, or proneuronal phenotypes [5]. Each of these numerous groups is characterized by its own course of the disease (different rates of tumor growth, sensitivity to therapy and, as a result, different patient survival). Thus, to achieve the best results in treatment, it is extremely important to correctly and timely determine the molecular phenotype of the tumor and, based on this, choose the drug that will be optimal for the treatment of each individual patient.

Previously, our and other groups obtained results indicating that the way of splicing of pre-mRNA molecules in tumor cells plays an important role in the formation of glioblastoma heterogeneity [6]. For example, the splicing type of Ras and CyclinD1 determines the rate of tumor cell proliferation [7], PKM alternative splicing changes the type of metabolism [8], and MDM4 and MDM2 protein isoforms regulate tumor sensitivity to therapy [9, 10]. However, the factors that cause differences in pre-mRNA splicing between tumor cells, the role of splicing changes in disease progression, and the mechanisms of the effect of splicing on the glioblastoma cell phenotype still remain obscure.

To identify the splicing regulator proteins most strongly associated with the main properties of low-grade gliomas (WHO grades II–III) and glioblastomas (grade IV), we decided to conduct a bioinformatics analysis of available RNA sequencing data from the TCGA database (The Cancer Genome Atlas), as well as to perform sequencing of RNAs isolated from tumor tissue samples and primary cultures of glioblastoma cells collected by our laboratory over the past 5 years.

We started our analysis by identifying the proteins involved in pre-mRNA splicing. Using the KEGG and NCBI databases, we created a list of 197 proteins that presumably affect splicing. It should be noted that this list is probably somewhat redundant, because, based on the analysis of protein structure and protein–protein interactions, only 50–150 proteins in the cell can directly regulate splicing [11, 12].

After analyzing the expression levels of spliceosomal protein genes in more than 150 patients with glioblastomas, we showed that all glioblastomas can be divided into several groups according to the expression of splicing factors (Fig. 1a). The first group primarily includes mesenchymal tumors (more than 75%) and is characterized by the worst prognosis in terms of patient survival (Fig. 1b). The second group consists mainly of tumors of proneuronal or neuronal phenotypes (more than 70%) and, on the contrary, is characterized by the longest life expectancy of patients. For the remaining samples at this stage of the work, we were unable to identify any striking distinctive features. Next, we performed transcriptome sequencing of primary cultures of glioblastoma cells (18 lines), human astrocytes (5 lines), and neural progenitors (3 lines). Figure 1c shows that all these groups of cells are well distinguishable from each other by the level of gene expression of spliceosomal proteins. In this experiment, individual clusters of proneuronal and mesenchymal cells, as well as a group containing poorly characterized lines were easily identified. These data are in good agreement with the results of our analysis of the TCGA database.

**Fig. 1.**
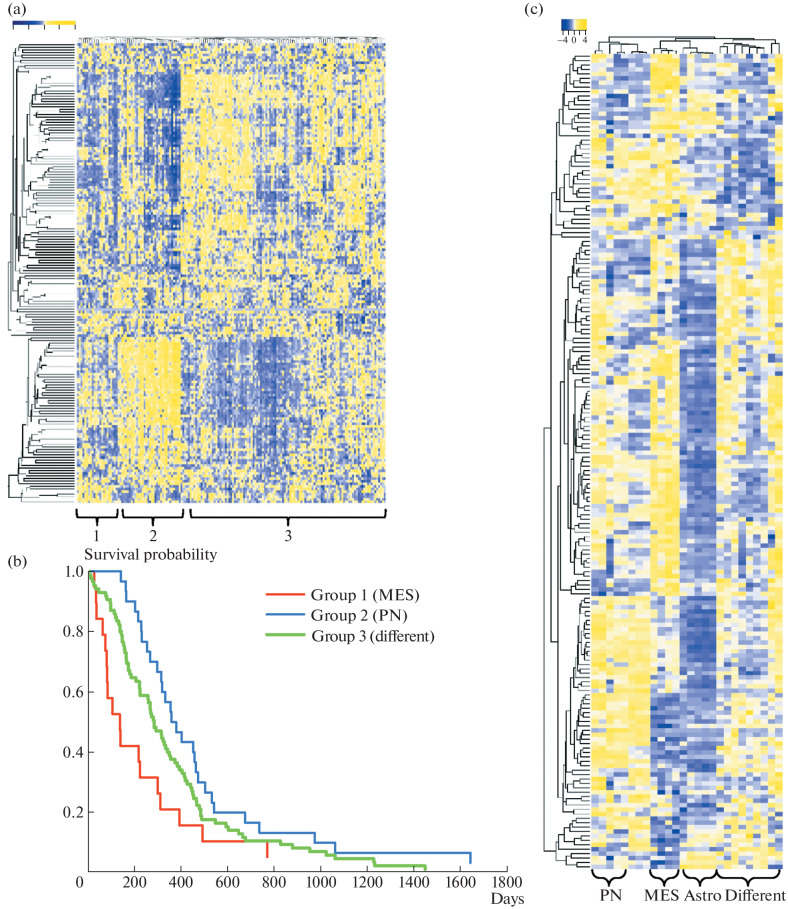
Association between expression of spliceosome genes and glioblastoma phenotype. (a) The heatmap demonstrating expression of spliceosomal genes; (b) Kaplan–Meier curves showing the overall survival of GBM patients divided into groups according to the expression of spliceosomal genes; (c) the heatmap reflecting spliceosomal gene expression in GBM sphere lines with different phenotypes (PN, MES, different) and human astrocytes (Astro).

To understand how the changes in spliceosomal gene expression that we observed affect the process of pre-mRNA splicing, we compared alternative splicing events detected by transcriptome sequencing of proneuronal and more aggressive mesenchymal primary glioblastoma cell cultures. In total, we found 3370 alternative splicing events that were statistically significantly different between the two groups of samples. These events affected important tumor-associated genes such as *MDM2*, *MDM4*, *FoxM1*, *hRAS*, *Notch1*, *ATM*, *TGM2*, *Drosha*, etc. Next, we divided all detected splicing changes into eight 8 types: (1) altered 3' splicing site, (2) altered 5' splicing site, (3) alternative mRNA end, (4) alternative mRNA start (strictly speaking, this change does not apply to splicing but results from the use of an alternative promoter), (5) single exon skip/add, (6) multiple exon skip/add, (7) mutually exclusive exons, and (8) intron retention (Fig. 2a). Analysis of gene enrichment in each of the eight types of splicing events showed that, for groups 1, 2, 3, 7, and 8, no significant enrichment in any genes was observed. However, for group 4, we found a significant enrichment in the genes responsible for cell adhesion, chemotaxis, and neuronal differentiation. Group 5 was enriched in the transcripts involved in DNA repair and cell cycle regulation, and group 6 was enriched in the genes encoding cell morphogenesis proteins (Fig. 2b).

**Fig. 2.**
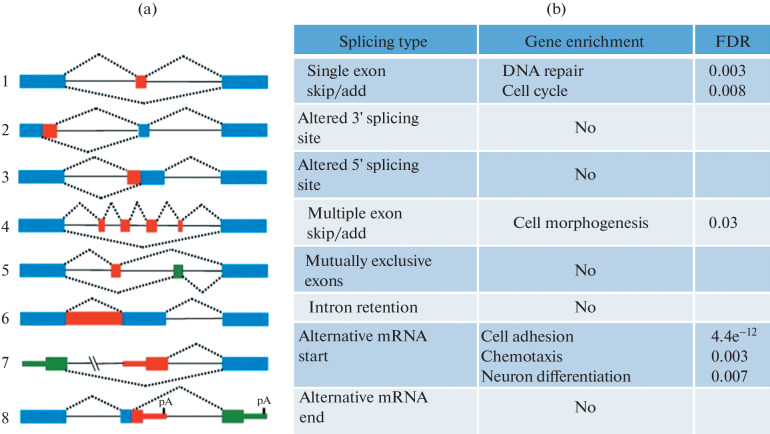
Differences in pre-mRNA splicing between proneural and mesenchymal glioblastoma cells. (a) The schematic picture of different types of alternative splicing events. (b) Enrichment analysis of the genes whose mRNAs are spliced differently in proneural and mesenchymal glioblastoma cells.

This result is in good agreement with the phenotypic differences between proneuronal and mesenchymal glioblastoma cells. For example, we have previously shown that mesenchymal cells are characterized by increased migration rate and invasiveness, greater resistance to DNA-damaging chemotherapeutic drugs, accelerated proliferation, and altered cell morphology [13]. Thus, it can be postulated that pre-mRNA splicing, indeed, determines some of the phenotypic features of glioblastoma cells.

After obtaining data on the expression of spliceosomal genes in tumors from different patients, we analyzed the levels of RNA splicing regulators within a single tumor. Previously, we demonstrated large phenotypic differences between cells from the center and periphery of glioblastoma [14]. For this reason, we compared the expression levels of splicing regulator genes in paired tumor tissue samples obtained from the edge and center of tumors from three different patients. After analyzing the RNA sequencing data, we showed that the central and peripheral parts of the tumor differ greatly in the expression profile of splicing factors. In accordance with this, the studied samples were clustered with each other but by the zone of glioblastoma they belonged to rather than by the patient from which they were obtained (Fig. 3a). It is also interesting to note that, according to our data, the diversity of spliceosomal proteins in the central zone of the tumor is significantly greater than in the cells infiltrating the normal brain.

**Fig. 3.**
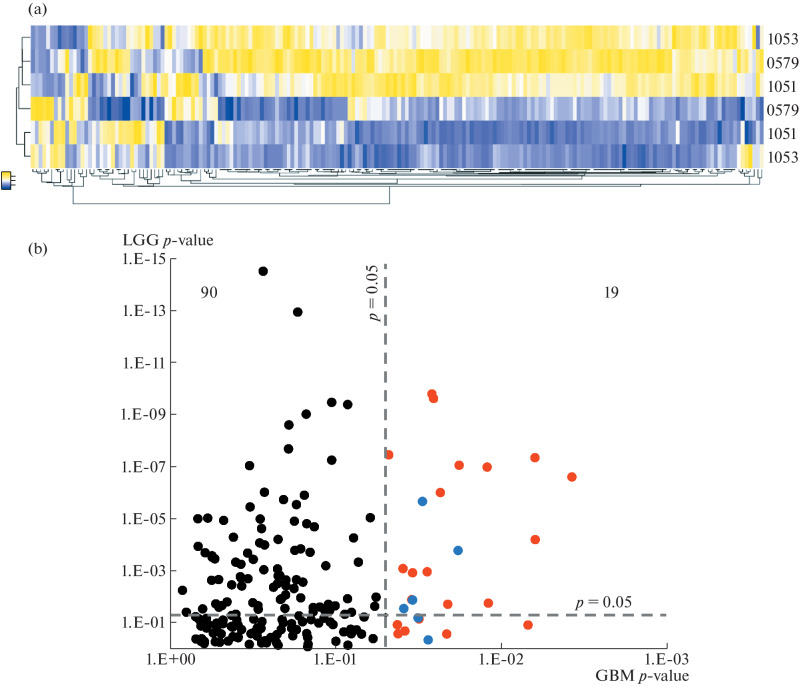
The effect of splicing regulator proteins on glioblastoma patient’s survival. (a) The heatmap demonstrating expression of spliceosomal genes in tumor tissue from the center (red) and from the periphery (blue) of glioblastoma from three different patients. (b) The diagram illustrating splicing factors, the expression of which is statistically significantly correlated with the survival rate of patients with glioblastoma and gliomas grade II–III. Red dots represent spliceosomal genes which low expression level correlates with poor GBM patients’ survival. Blue dots represent spliceosomal genes which high expression level correlates with poor GBM patients' survival.

Finally, we analyzed the TCGA database to identify the splicing factors whose expression is statistically significantly associated with survival in patients with grade II–III glioma and glioblastoma. Figure 3b shows that 27 spliceosomal genes whose expression can serve as a prognostic factor for patients were found for glioblastoma. Interestingly, for grade II–III glioma, the number of such genes was 4 times greater (109). In addition, it is worth noting that, for the majority of splicing factors (21 out of 27), the reduced expression of the corresponding gene was associated with poor patient survival. This result is in good agreement with the hypothesis that tumor and stem cells are characterized by a reduced level of pre-mRNA splicing control, which allows increasing the diversity of protein isoforms in the cell and, thus, maintain pluripotent properties. In addition, these data are an important confirmation of our previously published results that, in order to more effectively resist therapy, tumor cells use various mechanisms to reduce the level of spliceosomal proteins [15].

Summarizing the above results, it can be postulated that splicing-regulatory proteins play an extremely important role in determining the phenotype of glioblastoma cells and, theoretically, can be used to predict the course of the disease in patients with glioblastoma. However, obviously, further studies are required to identify the functions of specific spliceosomal proteins.
